# Morphological remodeling during recovery of the neuromuscular junction from terminal Schwann cell ablation in adult mice

**DOI:** 10.1038/s41598-020-67630-1

**Published:** 2020-07-07

**Authors:** Robert Louis Hastings, Michelle Mikesh, Young il Lee, Wesley J. Thompson

**Affiliations:** 1 Texas A&M Institute for Neuroscience, Texas A&M University, College Station, TX USA; 2 Department of Biology, Texas A&M University, College Station, TX USA; 3 Center for Biomedical Research Support (CBRS), University of Texas At Austin, Austin, TX USA

**Keywords:** Cell biology, Neuroscience

## Abstract

Schwann cells (SCs) are integral to the formation and function of the peripheral nervous system (PNS). Exemplifying their importance, the loss or dysfunction of SCs is a feature of a myriad of diseases and conditions that compromise the PNS. Thus, it remains essential to understand the rules that govern the proliferation, differentiation and reconnection of Schwann cells with peripheral axons. Here, we examined the consequences of locally and acutely ablating terminal Schwann cells (tSCs) at the adult mouse neuromuscular junction (NMJ) by using mice expressing diphtheria toxin receptor (DTR) preferentially in tSCs compared to myelinating SCs followed by local application of diphtheria toxin (DTX). After DTX application, tSCs died but, importantly and contrary to expectations, their associated motor axons did not fully degenerate. Within 3 weeks, tSCs returned and reestablished coverage of the synapse with increased numbers. Furthermore, the post-synaptic muscle fibers displayed increased distinct clusters of acetylcholine receptors and axon terminals exhibited numerous terminal varicosities. The lack of degeneration of bare motor axon terminals and the morphological remodeling that occurs upon the return of tSCs to the NMJ may have wider implications for the mechanisms governing tSC occupancy of the adult NMJ and for conditions that adversely affect tSCs.

## Introduction

Schwann cells (SCs), the glia of the peripheral nervous system (PNS), serve a variety of important roles^1–3^. Terminal Schwann cells (tSCs) are a specialized group of non-myelinating SCs that cap the axon terminal of α-motor neurons at the neuromuscular junction (NMJ)^4,5^. There is growing evidence that tSCs are affected in peripheral neuropathies, with some disease states resulting in tSC loss^6^. It is therefore important to have a better understanding of the roles of tSCs at the healthy NMJ, the consequences of tSC disorder and death, and how the nervous system recovers from tSC injury.

The NMJ, long used as a model synapse because of its large size and ease of accessibility, has three major components: the pre-synaptic motor axon terminal, the post-synaptic muscle fiber, and the tSCs, with evidence suggesting a fourth cell type, the kranocyte^7^. Using the advantages of the NMJ as a model synapse, experiments selectively ablating tSCs have offered insight into the normal functions of tSCs and the consequences of glial cell death at the NMJ. The first such study involved the use of a tSC specific antibody in frogs to activate the complement system, which caused the selective ablation of tSCs^8^. This study showed that in frogs, tSCs are not required for the acute function of the motor axon, but are necessary for the long-term integrity and maintenance of the NMJ. In addition, this study showed that when SCs were ablated in developing tadpoles, NMJs failed to form properly. In the following decades, a group of studies have utilized anti-ganglioside antibodies and a similar complement-mediated ablation of axons and tSCs in mice to mimic Guillain–Barré Syndrome^9^. Experiments that caused complement-mediated tSC ablation in young adult mice found that, similar to frogs, tSC death had no immediate impact on the neuromuscular function of the mammalian NMJ^10^.

Within the past decade, researchers have used the diphtheria toxin (DTX) system to systemically ablate SCs in mice. This system is mediated through a glia-specific Cre transgene that induces the expression of either the diphtheria toxin receptor (DTR) or the toxic A subunit of DTX. In a set of experiments on embryonic NMJs, developing motor axons degenerated without SCs and failed to form mature NMJs^11–13^, agreeing with the previous results from tadpoles and suggesting that SCs are necessary for synapse formation across vertebrate species. The diphtheria toxin system has also been used in adolescent (3–4 weeks old) mice to examine the consequences of systemic SC ablation. One such study observed SCs associated with low-threshold mechanoreceptors (LTMR) in the skin of mice^14^. After ablation, they found that axons degenerated without their associated SCs, but different subtypes of LTMR axon terminals degenerated at different rates. Another study examined adolescent mouse NMJs up to 6 days after systemic SC ablation, and observed that physiological deficits appear and portions of receptors lose axonal contact within the first week after SC injury^12^.

To date, studies involving the selective ablation of Schwann cells at peripheral axon terminals have observed and analyzed the short-term effects of SC loss on the synapse. As the next logical step, we sought to ablate tSCs at the NMJs of adult mice and track the recovery process after injury. To avoid the health problems that afflict a mouse after the systemic ablation of glia, which accompanies the injection of the DTR-expressing animals with DTX or Cre-mediated expression of the diphtheria toxin itself, we pursued a more localized DTX application in animals whose tSCs express DTR. To achieve a more localized ablation, we bathed a single sternomastoid (STM) muscle in DTX diluted in lactated Ringer’s. Using this method, there was widespread death of tSCs across the STM, but the mice still appeared healthy and behaved normally 3 weeks after DTX application.

Based on previous experiments of short-term tSC ablation, we expected exposed axon terminals to eventually degenerate to a pre-terminal node, leaving an endplate temporarily devoid of both tSCs and axon terminal. Thereafter, surviving SCs associated with the proximal stump of the axon would facilitate its regeneration back to the endplate. However, rather than withdrawing completely and regenerating to their NMJs, we found that when axons lost tSC coverage, few NMJs became completely denervated. By 3 weeks post-tSC ablation, NMJs were noticeably remodeled. Terminal Schwann cells had re-established their coverage of the motor axon terminals, but also increased in number and axon terminals had lost their smooth, continuous synaptic contacts and now displayed numerous, small varicosities attached by thin strings of axonal process. This appearance is strikingly similar to the “fragmented” NMJ: a phenotype common to several injury paradigms in which the pre- and post-synaptic components are remodeled to resemble beads on a string or grapes on a vine^15–23^. In keeping with recent observations proposing active roles for glia in synaptic remodeling^24–26^, in normal development, and in the maintenance of the NMJ, the results of this study suggest that tSC activity during recovery from selective injury involves a complex and active role for tSCs in the regeneration of the NMJ.

## Methods

### Animals

All experimental procedures were approved by Texas A&M University Institutional Animal Care and Use Committee and conducted in accordance with National Institutes of Health guidelines. Animals were sacrificed via intraperitoneal injection of Euthanasia-III Solution (Med-Pharmex).

Young adult (2–6 months old) mice on C57BL/6 background, with equal number of males and females in each group, were used for these experiments. All mice used in DTX ablation experiments possessed at minimum the plp1-Cre^ERT^ transgene, Jackson Lab (JAX) 005975^27^, and a transgenic cytoplasmic fluorescent protein, either the “Kosmos” (S100-GFP) transgene, JAX 005621^28^, which labels Schwann cells with cytoplasmic enhanced green fluorescent protein (GFP) under the S100 promoter, or the “Ai14” line, JAX 007914^29^, which labels cells with cytoplasmic tdTomato in a Cre dependent manner. The tdTomato expression pattern of tamoxifen-induced mice possessing plp1-Cre^ERT^ and Ai14 alleles was identical to the GFP expression pattern of the Kosmos transgene at the NMJ (see Results, Supplementary Figure S1). Some mice used in single-imaging experiments and all mice used in vital imaging experiments expressed the thy1-CFP transgene, which expresses cytoplasmic enhanced cyan fluorescent protein (CFP) in peripheral axons, JAX 003710^30^. Crucially, the difference between experimental animals (labeled “EXP”) and control animals (labeled “SHAM”) was that the EXP animals possessed the diphtheria toxin receptor (DTR) knock-in allele, JAX 007900^31^, while the SHAM animals lacked the diphtheria toxin receptor allele. EXP and SHAM mice were given identical treatments otherwise. Only mice that were heterozygous for the DTR knock-in were used for these experiments in order to minimize any potential experimental variation due to the copy number of DTR. During data analysis, it became apparent that NMJs 3 weeks post-tSC ablation displayed a phenotype similar to axonal neuregulin 1 type III over-expression. To gain insight into the possible involvement of the neuregulin 1 (NRG1) signaling pathway, we bred EXP mice with a constitutive NRG1 knockout mouse line^32^. This breeding generated “EXP, NRG1 +/−” mice, which had the same genotype as EXP mice in addition to being hemizygous for the NRG1 null allele.

### Tamoxifen treatment

Mice used in these experiments, whether EXP or SHAM, received a full regimen of tamoxifen (TMX). Injectable TMX solution was made by first dissolving powdered TMX (Sigma-Aldrich T5648) in 100% ethanol at a concentration of 60 mg/mL. The ethanol-TMX solution was then mixed with sunflower seed oil (Sigma-Aldrich S5007) and the ethanol was evaporated off the top. The final concentration of the injectable TMX solution was 20 mg/mL tamoxifen in sunflower seed oil. Cre recombinase activity was induced in mice by injecting them with 100 µg TMX/g body weight once a day for 5 consecutive days.

### Diphtheria toxin application

Mice were anesthetized in an incubation chamber with 4% isoflurane and then maintained at 1.5% isoflurane with 1 L/min oxygen flow rate. Depilatory cream was used to remove the fur on the ventral side of the neck, an antiseptic 10% iodine solution was applied to the skin, and a midline incision was made from the mandible to the sternum. The submandibular glands were pulled aside and the sternomastoid (STM) muscle was exposed using blunt dissection. The exposed STM muscle was bathed in 10 μg/mL diphtheria toxin (DTX) diluted in lactated Ringer’s for five minutes. For vital imaging experiments, DTX was applied to the muscle after the initial imaging session. The muscle was washed thoroughly with lactated Ringer’s, and the wound was closed with nylon suture and a 6–0 needle.

### Vital imaging

Experiments involving in vivo imaging were conducted as previously described^33^. Briefly, the animals were anesthetized with a ketamine/xylazine cocktail (65 mg/kg body weight ketamine, 5 mg/kg body weight xylazine) and placed on a stage in a supine position. Depilatory cream was used to remove the fur on the ventral side of the neck, antiseptic iodine solution was applied to the skin, and a midline incision was made from the mandible to the sternum. An endotracheal tube was inserted into the mouse’s trachea and connected to a ventilator (Harvard Instruments, Model 683). The submandibular glands were pulled aside and the sternomastoid (STM) muscle was exposed using blunt dissection. The exposed STM muscle was bathed with 2 μg/mL Alexa Fluor 488, 555, or 647-tagged α-bungarotoxin (Invitrogen), depending on what fluorescent channel was not being used to image tSCs, dissolved in sterile lactated Ringer’s for five minutes to allow for the visualization of the post-synaptic acetylcholine receptors. This α-bungarotoxin (BTX) stain blocks acetylcholine receptors at a level below that which is necessary to affect transmission. Any excess BTX was removed by several washes with lactated Ringer’s solution and the STM was slightly elevated using a small metal ring attached to a micromanipulator. No coverslips were used in these in vivo experiments. The neuromuscular junctions (NMJs) of the STM were then imaged with IP Lab software using a Photometrics Cool SNAP HQ camera mounted on an Olympus BX51WI epifluorescence scope with a 20×, 0.95 numerical aperture water immersion objective. Images of slightly different focal planes were stitched together using the Photomerge Faces feature of Photoshop Elements 6 to create one image. Animals were vitally imaged three times, 1 week apart: an initial imaging session before DTX application, at 1 week post-DTX, and at two weeks post-DTX. The animals were sacrificed at three weeks post-DTX.

### Fluorescence immunohistochemistry

Animals were sacrificed, transcardially perfused with phosphate buffered saline (PBS), and the muscles were immediately dissected, pinned in a dish, fixed in 4% paraformaldehyde (PFA) in PBS for 20 min, and washed 3 times for 5 min each in PBS. The muscles were then stained and prepped for whole mount, which was achieved by pinning the whole muscle into a sylgard-lined dish and using microsnips and forceps to peel the top layer of muscle fibers away from the surface of the muscle. The resulting “fillets” were mounted on a slide and coverslipped. Fixed tissue was imaged using a Leica DMRX epifluorescence microscope, a Zeiss LSM 780 confocal microscope at the Texas A&M University College of Veterinary Medicine & Biomedical Sciences Image Analysis Lab, or a Leica S5 confocal microscope at the University of Texas at Austin.

Primary antibodies used in these experiments were as follows: a combination of SV2 and 2H3 (Developmental Studies Hybridoma Bank, antibody registration numbers 2315387 and 2314897, respectively) for labeling α-motor axon terminals and axons, anti-Tubulin β-3 (Tuj1, Biolegend 802001) for labeling all axons in the muscle tissue, anti-tyrosine hydroxylase (Millipore, mab318) to label sympathetic axons in the muscle tissue, anti-S100 (Dako, Z0311) to label all SCs, and anti-Human HB-EGF (R&D Systems, AF-259-NA) to label cells expressing diphtheria toxin receptor. Secondary antibodies used in these experiments were as follows: goat anti-mouse IgG1 conjugated to either Alexa Fluor 488 (Life Technologies A-21121), Alexa Fluor 555 (Life Technologies A-21127), or Alexa Fluor 647 (Life Technologies A-21240), goat anti-rabbit FITC (Cappel 55661), donkey anti-goat Alexa 488 (Jackson ImmunoResearch), donkey anti-rabbit Alexa 647 (Jackson ImmunoResearch).

### Electron microscopy

Electron microscopy procedures are identical to those described previously^24,25^. Briefly, animals were sacrificed and transcardially perfused with 2% Paraformaldehyde with 3% Gluteraldehyde in 0.1 M Sodium Cacodylate buffer. Muscles were dissected and pinned into sylgard dishes and allowed to fix overnight, then washed and *en bloc* stained with 1% Potassium Ferrocyanide with 1% Osmium Tetroxide in buffer, washed in water, and stained in 1% Uranyl Acetate followed by water. Muscles were dehydrated through graded alcohols to absolute acetone, infiltrated with Epon 812, and polymerized. Thick sections (0.5 µm) were collected to identify regions of interest prior to collecting thin sections (70 nm) and mounting to formvar coated Synaptek grids. Images were collected on a Technai Spririt BioTwin at 80 kV using an AMT Advantage HR digital camera.

### Data collection and analysis

Data were collected by imaging a “fillet” of each STM and selecting NMJs that were near the surface and in an *en face* orientation. Measurements were limited to surface NMJs because they had best exposure to the DTX and image quality drops when depth of imaging increases. All measurements were taken by imaging these surface NMJs using iVision software on a Leica DMRX epifluorescence microscope with a 40×, 1.00 NA oil objective. Each variable was collected for each NMJ.

The numerical variables collected were number of tSCs, number of terminal boutons, number of acetylcholine receptor (AChR) islands, and junctional area. Number of tSCs was counted by finding cell bodies using the transgenic fluorescent label and confirming the presence of a nucleus using DAPI stain. “Terminal bouton” in this study refers to a varicosity of the axon terminal that is connected to the rest of the axon terminal by a thin process. These boutons vary in size, but their key defining features is their separation from the rest of the axon by a narrow, sometimes indiscernible, piece of axon culminating in a varicosity. The number of AChR islands was measured by manually counting distinct islands of BTX stain. The junctional area was calculated by iVision after drawing a perimeter around the BTX stain.

The categorical variables collected were Schwann cell coverage, innervation status, presence of terminal sprouts, and presence of central myonuclei. Schwann cell coverage was measured as “fully covered,” “partially bare,” or “fully bare” depending on the amount of Schwann cell fluorescent signal that overlaid the AChR stain. NMJs were classified as “partially bare” if a portion of the AChR stain did not have corresponding SC label, i.e. greater than 0%, but less than 100% coverage of the AChRs by SC processes. Innervation status was measured as “innervated,” “partially innervated,” or “denervated” depending on how completely the axonal fluorescent signal overlaid the AChR stain. NMJs were classified as “partially innervated” if a portion of the AChR stain did not have corresponding axon terminal label, i.e. greater than 0%, but less than 100% coverage of the AChRs by the axon terminal. Sprouting was measured by determining if Schwann cell signal or axonal signal exceeded the boundaries of the AChR stain, and each sprout was given a classification of sprouts with only Schwann cell signal, sprouts with Schwann cell and axonal signal, or sprouts that have axonal signal but no Schwann cell signal. The central myonuclei variable, which was recorded to determine the level of myofiber damage in the tissue, was given a binary “Yes” or “No” depending on whether the muscle fiber of the NMJ being measured had a string of central nuclei running through it near the endplate area.

All statistical analyses were run on GraphPad Prism 8 and all graphs were made in Microsoft Excel. Equal variances were not assumed, so Welch’s t-tests were run when comparing two numerical variables. For each categorical variable, the expected value of at least one of the conditions was too low to run a standard Chi Square test for independence, so a Fisher Exact test was run instead. In order to fulfill the requirements of the Fisher Exact test, the expected wild-type (WT) conditions (fully covered axon terminals, fully innervated endplates, no terminal sprouts, no central nuclei) were compared with the pooled values of the other conditions in the variable. The resulting test compared the distribution of expected wild type condition with all other conditions between experimental and sham animals. All values are expressed as mean ± standard error of the mean.

## Results

### The plp1-Cre^ERT^ transgene drives DTR expression in terminal Schwann cells

**Figure 1:**
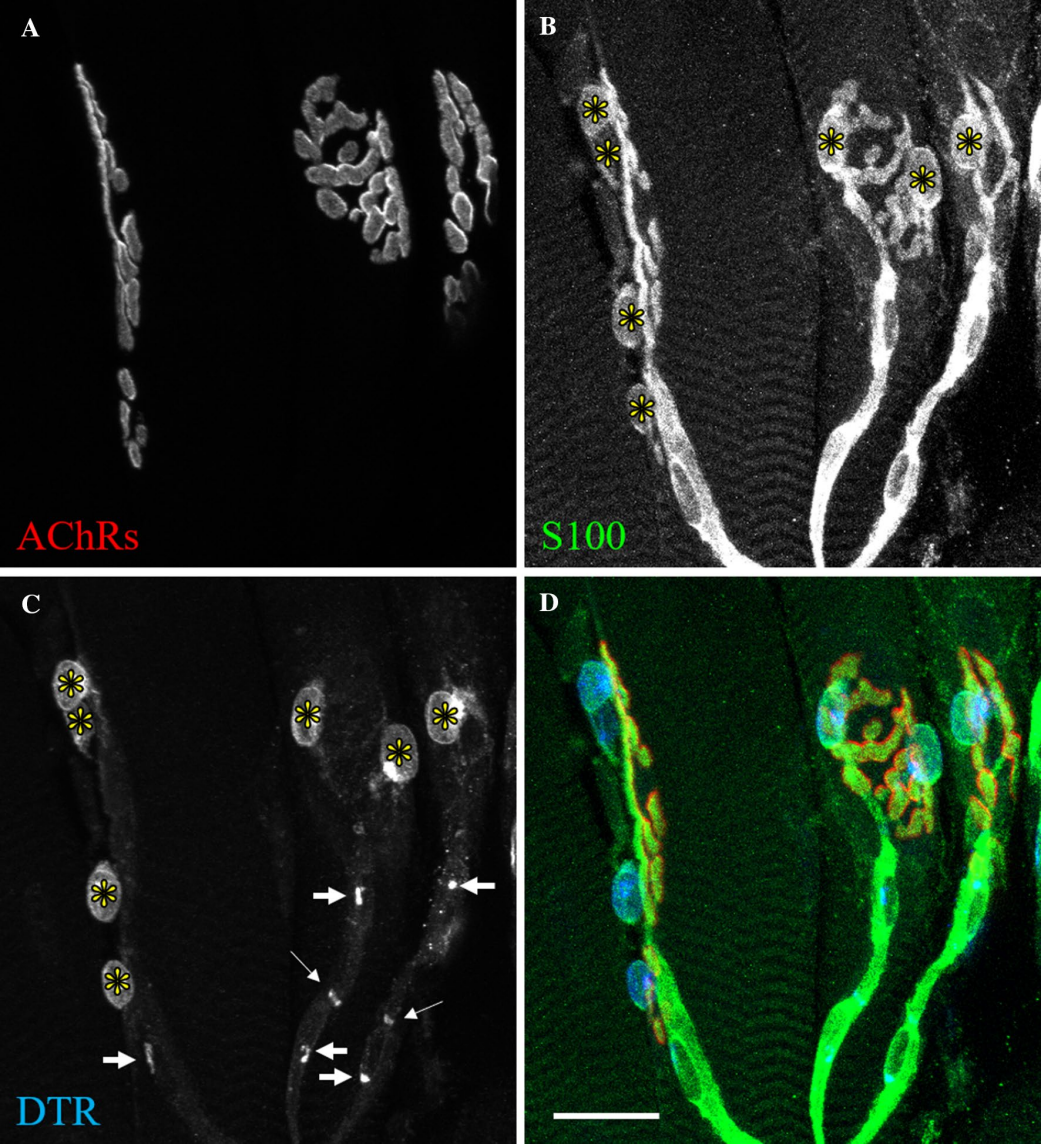
The plp1-Cre^ERT^ transgene preferentially drives expression of DTR in tSCs at the NMJ. Young adult NMJs expressing plp1-Cre^ERT^ and DTR transgenes were stained with BTX to label AChRs (**A**) and for S100 to label tSCs (asterisks) and myelinating SCs (**B**). DTR stain is robust in the soma of each tSC (asterisks), but weak in their processes (**C**). In myelinating SCs, DTR stain seems to be concentrated in paranodal microvilli (thin arrows, **C**) and in small spots in each nucleus (thick arrows, **C**). (**D**) Composite image of **A**–**C**. Fiji and Microsoft Powerpoint were used to generate this figure. Scale bar 20 µm.

The plp1-Cre^ERT^ transgene was designed for use in central nervous system oligodendrocytes, and detailed characterization of this transgene at the neuromuscular level of adult mice had yet to be carried out^27^. To determine the expression pattern of the plp1-Cre^ERT^ driven DTR construct, we stained NMJs of tamoxifen-treated mice carrying the plp1-Cre^ERT^ and DTR transgenes, identified by BTX stain (Fig. 1A), with an anti-S100 antibody, to stain all SCs (Fig. 1B), and an anti-DTR antibody (Fig. 1C). The DTR antibody exhibited strong staining in the soma of each tSC, but weak staining in their processes (Fig. 1D). In each tSC and myelinating SC soma, there appeared to be a spot of more intense staining, which may be an area in which the protein is being processed, such as the endoplasmic reticulum. In myelinating SCs, the DTR stain was scant and seemed to be most concentrated at the paranodes of myelinating SCs, possibly due to hinderance of trafficking a foreign protein, whose expression is induced well after myelin sheath formation, to the outer membrane that is tightly associated with the myelin sheath. This is consistent with a previous report, which showed that DTR expression in myelinating SCs was limited to the paranodal microvilli, and that DTX injection did not affect sciatic nerve compound action potentials, suggesting a limited susceptibility of myelinating SCs to DTX ablation^34^. These results, taken together with our observations that myelinating SCs tended to remain after tSCs were ablated, suggest that the expression of DTR was more robust in tSCs compared to myelinating SCs, which may have led to a greater susceptibility of tSCs to the acute DTX application.

To determine the general expression of the transgene in muscle tissue, we crossed the plp1-Cre^ERT^ line with the Ai14 reporter line to express cytoplasmic tdTomato label upon Cre-mediated excision of a stop codon. When Cre expression was fully induced with tamoxifen, we found that the fluorescent reporter faithfully labeled both myelinating SCs and tSCs at the NMJ (Supplementary Figure S1). Sham animals used in this study had the expected average number of tSCs per young adult sternomastoid (STM) NMJ (3.51 ± 0.08) and the number of tSCs at individual NMJs moderately correlated with junctional area (r = 0.61, r^2^ = 0.36, p < 0.0001), consistent with past literature^35,36^. The plp1-Cre^ERT^ transgene also drives Cre activity in Schwann cells besides those associated with α-motor axons, including those far away from the endplate band (Supplementary Figure S1) and SCs associated with sympathetic axons (Supplementary Figure S1). Although they were not analyzed in this study, these extra-junctional Schwann cells would have been ablated in each of the following experiments along with the Schwann cells of the α-motor system.

### Terminal SC number and coverage gradually increase after ablation

**Figure 2:**
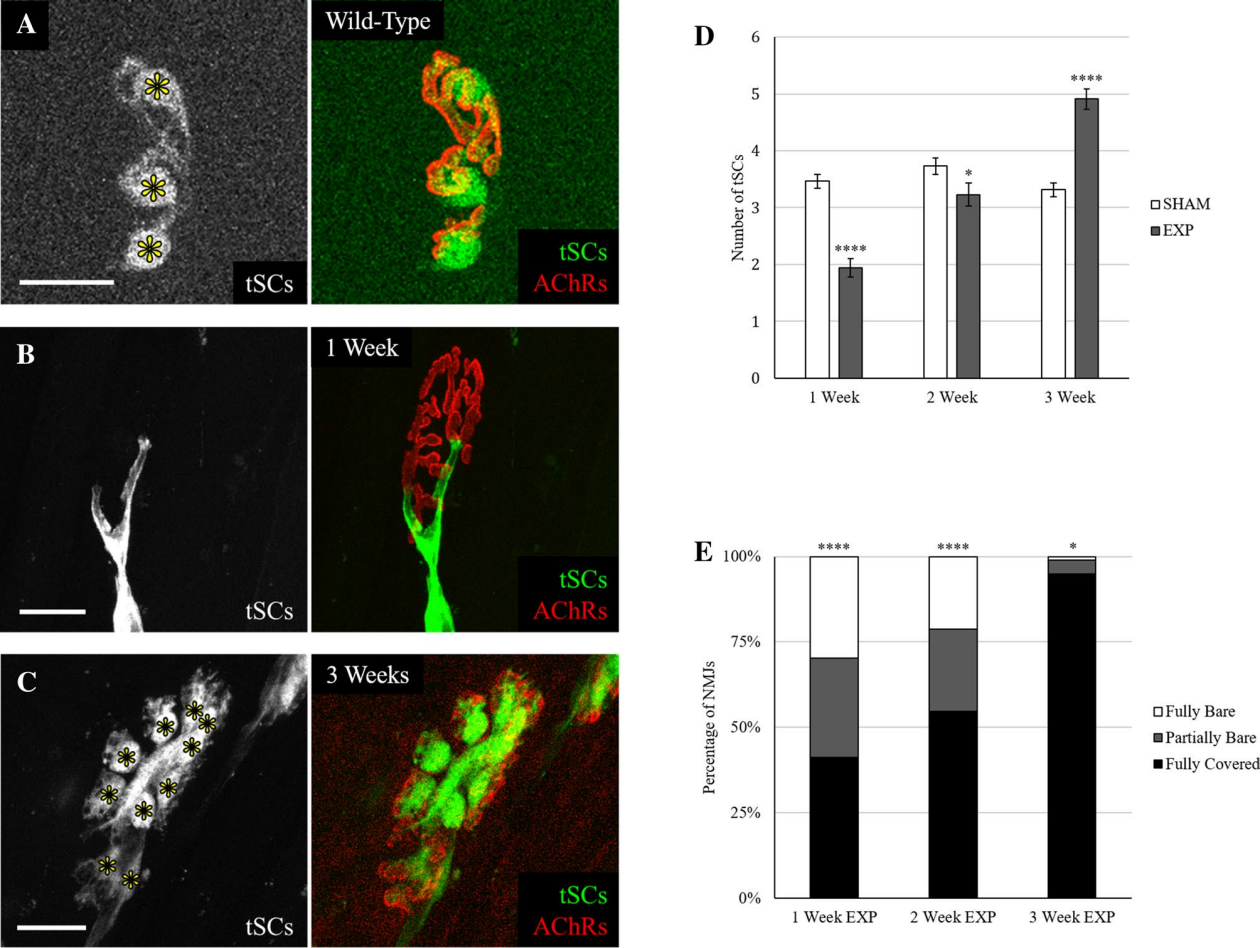
Terminal Schwann cells were ablated 1 week after DTX treatment and returned within 3 weeks. For each set of images, tSCs, denoted by asterisks, express cytoplasmic tdTomato (pseudo-colored green) and overlay their corresponding AChRs (pseudo-colored red) in the composite. (**A**) Wild-type neuromuscular junction. Three tSCs completely cover the AChR stain. (**B**) One week post-tSC ablation. All tSCs have been ablated, but some SC signal remains along the axon. (**C**) Three weeks post-tSC ablation. Ten tSCs completely overlay the AChR stain. (**D**) Average number of tSCs per NMJ in each group. At 1 week post-tSC ablation, 34% of all experimental NMJs had zero tSCs. Welch’s t-test was used to compare sham to experimental. (**E**) Percentage of NMJs that had full tSC coverage, were partially bare, or were fully bare. Each sham group showed > 98% of NMJs having full tSC coverage. Fisher Exact test was used to compare sham to experimental. n = 6 or 7 mice for each condition, 15–30 NMJs per mouse. Values expressed as mean ± SEM. Sham groups did not differ from other sham groups at any time point. Asterisks indicate significant difference from the corresponding sham group. *p < .05; ****p < .0001. Fiji and Microsoft Powerpoint were used to generate this figure. All scale bars 20 µm.

The average number of tSCs per NMJ is predictable for a given muscle, and adult STM muscles consistently average 3.5 tSCs per NMJ (Fig. 2A). At 1 week post-DTX (Fig. 2B), the experimental animals showed a drop in the average number of tSCs per NMJ to 1.9 ± 0.16. By 2 weeks, tSC number per NMJ had recovered to 3.2 ± 0.20, an average that was statistically less (p = 0.04) than sham controls. Between 2 and 3 weeks (Fig. 2C), however, tSC number per NMJ increased significantly, surpassing wild-type (WT) parameters to an average of 4.9 ± 0.18 tSCs per NMJ (Fig. 2D). The drop in tSC number at 1 week makes intuitive sense, because tSCs are being ablated in that time. It also stands to reason that the tSCs would steadily increase in number as the NMJ recovers from the injury between 1 and 2 weeks, however, it was surprising that the tSCs, as they re-populate the once abandoned NMJ, increased in number to the point that NMJs had significantly more tSCs than WT mice.

In WT animals, all NMJs are completely covered by tSC processes. One week after tSC ablation, however, NMJs showed a striking decrease in tSC coverage with just 41% of NMJs in experimental animals exhibiting axon terminals fully covered by tSC processes (Fig. 2E). Furthermore, 30% were entirely devoid of tSC coverage, leaving 29% with partial coverage. Through 2 and 3 weeks post-tSC ablation, the coverage of the endplate gradually recovered towards WT levels. At 2 weeks, 55% of NMJs were fully covered, 21% were fully bare, and 24% were partially bare. At 3 weeks, 95% of NMJs were fully covered, 1% were fully bare, and 4% were partially bare. Additionally, as tSCs re-established terminal coverage, tSC sprouts did not extend beyond the boundaries of the junctions more than in sham controls (Supplementary Figure S2). There were rare occasions in the sham controls (two in each time point) where an NMJ had partial tSC coverage, and in each of these cases the pattern of tSC label (two–three tSCs labeled with a “bare” spot the expected size of a single tSC territory) suggested that this was due to incomplete recombination of the Cre-inducible reporter in a single tSC. “Partially bare” terminals were never seen in sham animals when imaging with the “Kosmos” transgene, which labels all tSCs with constitutive expression of cytoplasmic GFP under the control of the S100 promoter. These results indicate that DTX-induced tSC death depletes tSC coverage of axon terminals, and that tSC coverage gradually increases over 3 weeks of recovery.

### Axon terminals remain at the synapse and are remodeled during the 3 weeks following tSC ablation

**Figure 3:**
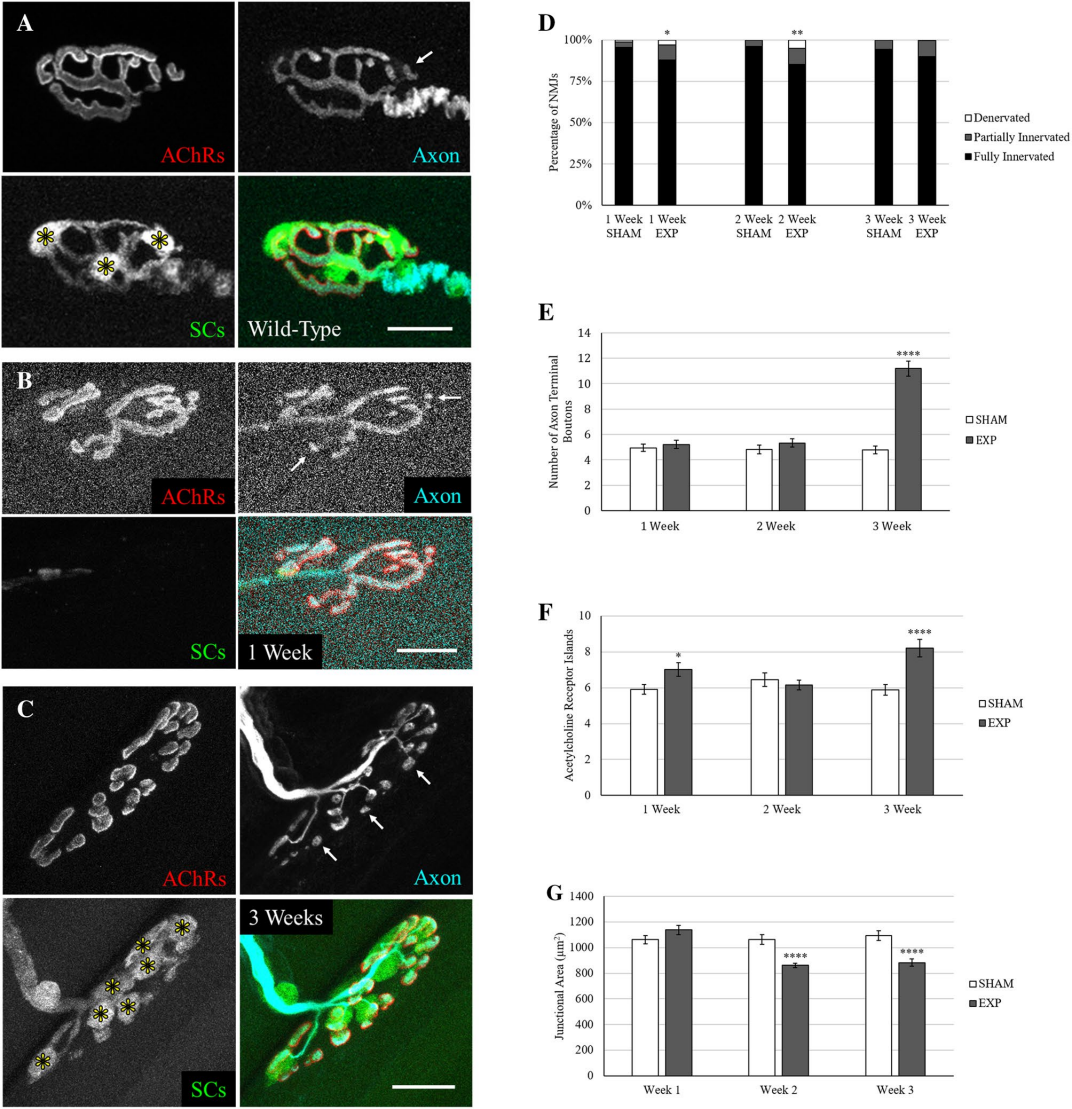
Morphological changes to the axon terminal and acetylcholine receptors after tSC ablation. For each set of images, arrows point to examples of varicose terminal boutons in the axon channel and asterisks denote tSC bodies in the SC channel. (**A**) Wild-type neuromuscular junction of a mouse expressing thy1-CFP transgene in α-motor axons and tdTomato in SCs. (**B**) One week post-tSC ablation. The motor axon and SCs were transgenically labeled with CFP and tdTomato, respectively. Note that the natural overlying tSC label is entirely missing, but the AChRs remain fully innervated. (**C**) Three weeks post-tSC ablation. SCs were transgenically labeled with GFP, the axon was stained with 2H3 and SV2 primary antibodies. Note the numerous tSC bodies and varicose terminal boutons at the junction. (**D**) Innervation of NMJs. Partially innervated NMJs are those with areas of AChRs with no corresponding axon terminal and fully denervated NMJs are those with no axon overlying the AChR stain. Fisher Exact test was used to compare sham to experimental. (**E**) Number of axon terminal boutons, or synaptic varicosities attached to the rest of the axon terminal by a thin process. Welch’s t-test was used to compare sham to experimental. (**F**) Average number of distinct islands of BTX-stained AChR aggregates per NMJ. Welch’s t-test was used to compare sham to experimental. (**G**) Average junctional area per NMJ, calculated from a perimeter drawn around the BTX stain. Welch’s t-test was used to compare sham to experimental. n = 6 or 7 mice for each condition, 15–30 NMJs per mouse. Values expressed as mean ± SEM. Sham groups did not differ from other sham groups at any time point. Asterisks indicate significant difference from the corresponding sham group. Fiji and Microsoft Powerpoint were used to generate this figure. *p < .05; **p < .01; ****p < .0001.

Based on previous research that showed axon terminals begin to degenerate after SC ablation^8,14^, we hypothesized that α-motor axons in the mouse would quickly degenerate after a week without tSC coverage. Surprisingly, a large majority of NMJs remained innervated (Fig. 3A, Fig. 3B, Fig. 3C) throughout the depletion and return of the tSCs (Fig. 3D), and repeated vital imaging of individual NMJs confirmed that the motor axons remain largely unchanged from their original morphology one week after tSC loss (Supplementary Figure S3). The fact that there are significantly more partially innervated or denervated NMJs, i.e. AChRs without corresponding pre-synaptic axon terminal, at 1 week post-tSC ablation agrees with a recent study ablating SCs in adolescent mice^12^. This significant difference in innervation extends to the 2 week time point but, crucially, at each time point more than 85% of NMJs are fully innervated in the experimental animals (Fig. 3D), despite the fact that only 41% of axon terminals maintained full tSC coverage 1 week after ablation (Fig. 2E). Although the large majority of NMJs were innervated at each time point after tSC ablation, the axon terminals displayed a striking feature at 3 weeks not present at 2 weeks: the existence of numerous terminal boutons, or extremely thin branches of axon culminating in a varicosity overlying the AChRs (Fig. 3C). These terminal boutons exist naturally in an adult mouse, but in small numbers (4.8 ± 0.18 per sham NMJ). Between 2 and 3 weeks post-tSC ablation, the number of terminal boutons increased to 11.2 ± 0.59 per NMJ. As shown in Fig. 3E, the tSC ablated axon terminal boutons are indistinguishable from sham controls until the remodeling event that occurs between 2 and 3 weeks.

### AChR islands increase and junctional area decreases after tSC ablation

The post-synaptic apparatus of the NMJ exhibits remarkable plasticity when a cellular component is injured. The fragmented phenotype we observed at 3 weeks is classically known as a consequence of myofiber damage^16^. In order to rule out that fragmentation was caused by incidental myofiber damage during surgery, we observed whether the myofiber associated with each NMJ had a chain of central myonuclei, a classic biomarker of myofiber regeneration^37^, within the vicinity of the junction and found no significant difference between tSC ablated NMJs and sham controls at any time point (Supplementary Figure S4). In order to assess the effect of selective tSC injury on the post-synaptic receptors, we counted individual islands of BTX stain and measured the junctional area of each NMJ.

Sham controls had a collective average of 6.1 ± 0.18 distinct AChR islands (Fig. 3F) per NMJ, which falls in line with previous literature analyzing the young adult STM muscle^21^. One week after tSC ablation, there was a statistically significant increase in the average number of AChR islands to 7.0 ± 0.37, which agrees with results from a recent study ablating SCs in adolescent mice^12^. Experimental animals were indistinct from sham controls at 2 weeks, but at 3 weeks there was a large statistically significant difference between sham controls and experimental animals, which averaged 8.2 ± 0.49 distinct AChR islands per NMJ. At the 1 week time point, the junctional area was not different between experimental and sham animals with 1,061 ± 33 µm^2^ per NMJ (Fig. 3G). At 2 weeks, the average junctional area per NMJ fell significantly to 861 ± 18 µm^2^ in experimental animals and remained at that level (883 ± 28 µm^2^) through three weeks.

### Terminal SCs intercalate beneath and phagocytose axon terminals during NMJ recovery after tSC ablation

**Figure 4:**
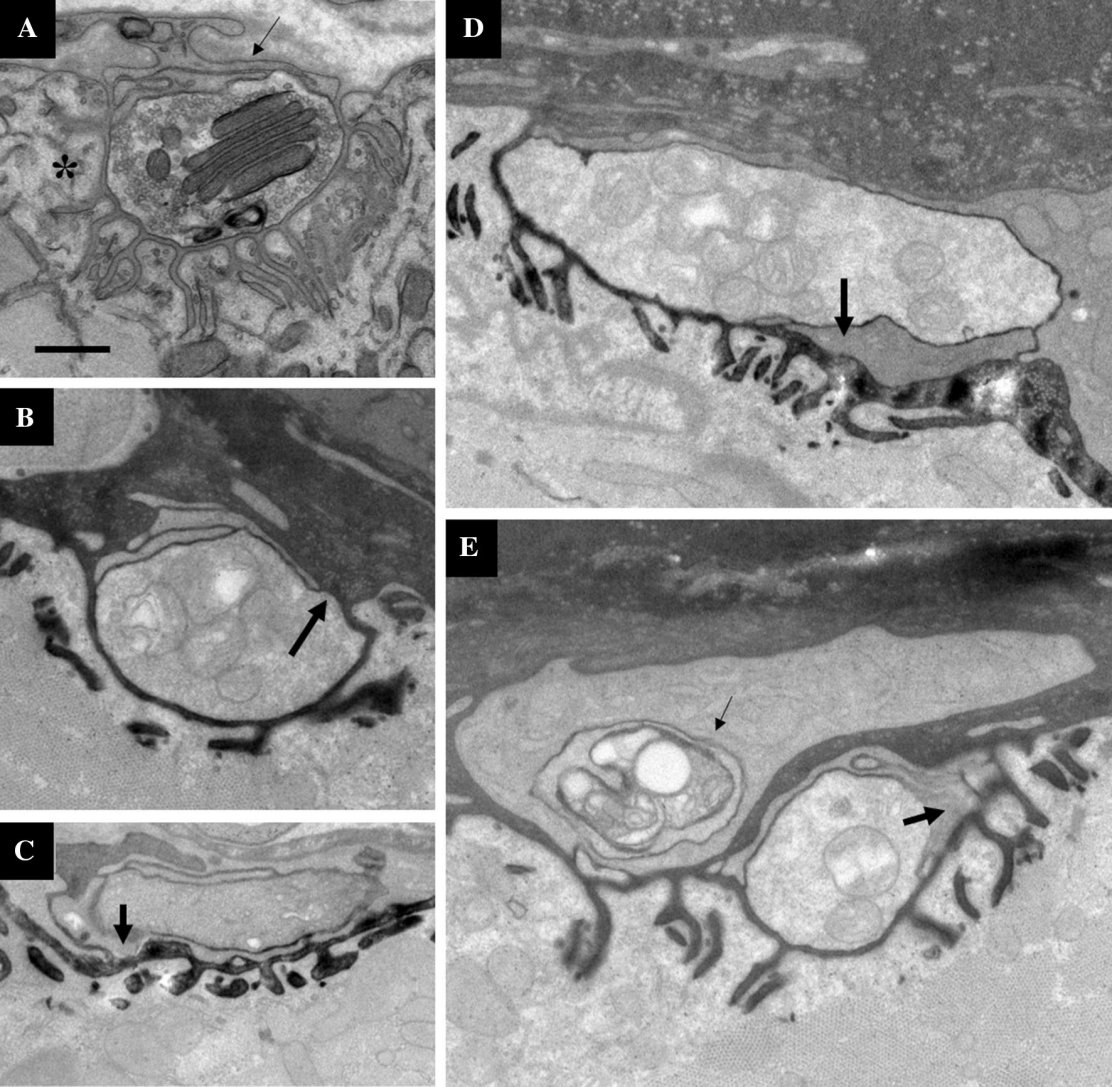
Electron micrographs of a WT NMJ (**A**) and NMJs from a 2 week post-tSC ablation animal (**B**–**E**). (**A**) Young adult WT mouse NMJ. Asterisk denotes post-synaptic muscle fiber and thin arrow denotes overlying tSC. (**B**) A portion of the axon terminal is without tSC coverage (thick arrow). (**C**) Terminal Schwann cell process completely separates axon terminal from post-synaptic gutter (thick arrow). (**D**) Terminal Schwann cell process partially separating axon terminal from post-synaptic gutter (thick arrow). (**E**) Terminal Schwann cell phagocytosing a piece of axon terminal (thin arrow) and another process separating the axon from the secondary folds (thick arrow). All panels are from different NMJs, except for **B** and **E**, which are different parts of the same NMJ. The dark appearance of the secondary folds in **B**–**E** are an artefact and not the result of specific staining. Microsoft Powerpoint was used to generate this figure. Scale bar 500 nm, applies for all panels.

Ultrastructural analysis of tSCs have previously revealed active roles in remodeling the NMJ that are not discernable at the resolution of light microscopy^24,25^. Therefore, to further explore the activity of tSCs during the remodeling process occurring between 2 and 3 weeks post-tSC ablation, we observed electron micrographs of an experimental animal 2 weeks post-tSC ablation. At the ultrastructural level, tSCs exhibited abnormal phenotypes 2 weeks after ablation (Fig. 4). Figure 4A shows a representative NMJ from a young adult WT mouse. The axon terminal is in close contact with the post-synaptic muscle fiber (asterisk) and all of the secondary folds while the tSC (thin arrow) completely caps the axon terminal and extends only slight processes in between the terminal and the muscle fiber. Figures 4B–E show NMJs 2 weeks after tSC ablation. Figure 4B shows a piece of the axon terminal that does not have tSC coverage (thick arrow), possibly indicating that this axon terminal is partially bare. Figures 4C–E show a tSC extending processes in between the axon terminal and the secondary folds (thick arrows), and in the case of Fig. 4C, completely wrapping the axon terminal. Figure 4E shows a tSC in the process of phagocytosing a piece of axon terminal (thin arrow). These observations suggest that the tSCs are actively altering NMJ morphology during recovery of the junction from tSC ablation.

### NRG1 signaling pathway mediates increase in tSC and terminal bouton number at 3 weeks post-tSC ablation

**Figure 5:**
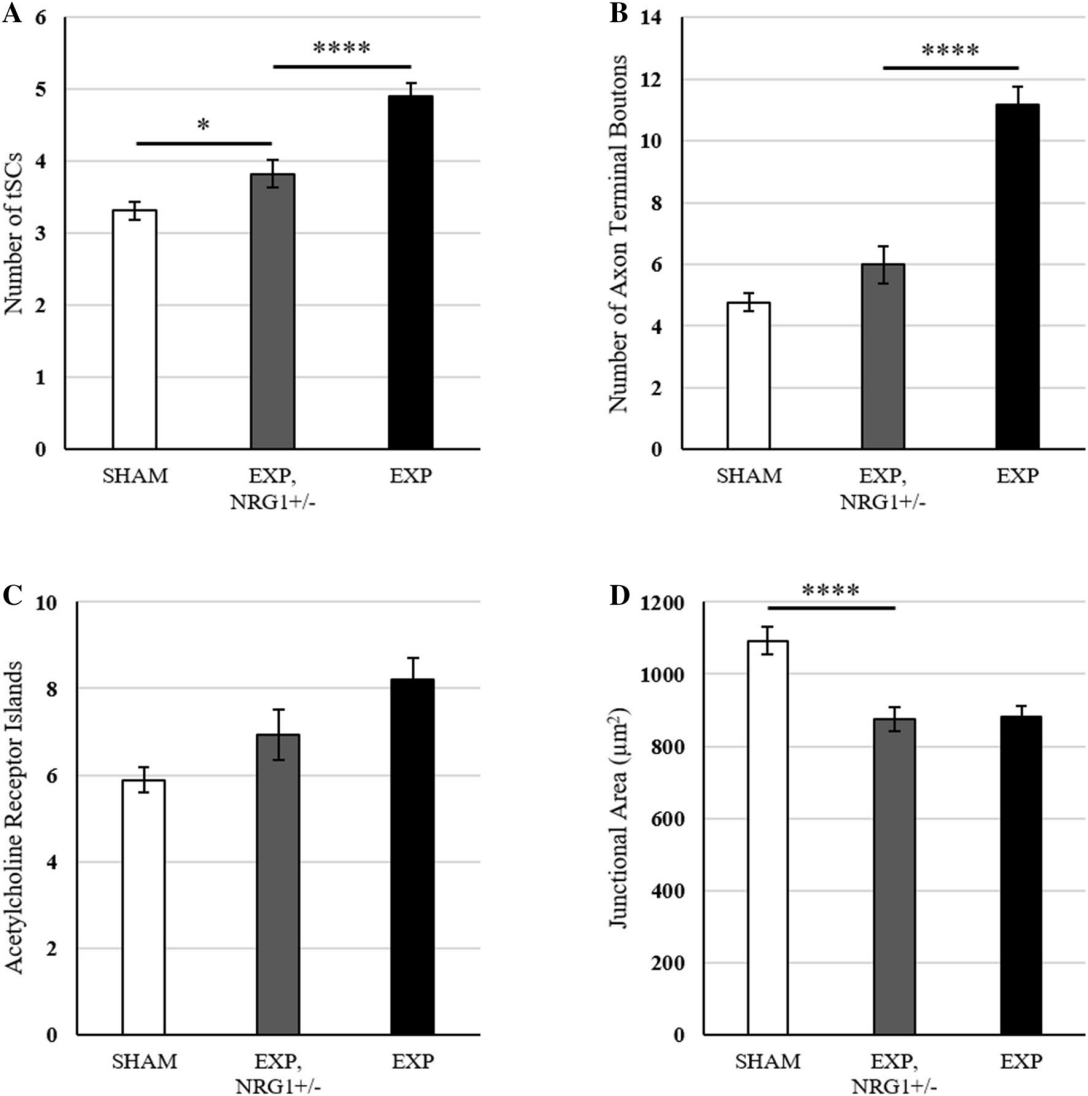
Morphological changes 3 weeks post-tSC ablation in EXP, NRG1 +/− mice compared to SHAM animals and EXP (DTR expressing, full NRG1 signaling) animals. (**A**) The average number of tSCs at EXP, NRG1 +/− NMJs was significantly different from both SHAM and EXP mice. (**B**) The mean number of axon terminal boutons at EXP, NRG1 +/− NMJs was not different than SHAM mice. (**C**) The average number of AChR islands of EXP, NRG1 +/− NMJs was not statistically different than either EXP or SHAM mice. (**D**) The mean junctional area of EXP, NRG1 +/− mouse NMJs was significantly different from SHAM mice, but not different from EXP mice. Welch’s t-test was used to compare each group. Data for SHAM and EXP animals were taken from Figs. 2 and 3. For EXP, NRG1 +/− mice, n = 4 mice, 15–30 NMJs per mouse. Values are expressed as mean ± SEM. Asterisks indicate significant difference between groups connected by a bar. Statistical significance between SHAM and EXP groups is depicted in Figs. 2 and 3. *p < .05; ****p < .0001.

The NMJ morphology seen in this study 3 weeks after tSC ablation was strikingly similar to that of mice with transgenic axonal neuregulin 1, type III (NRG1-III) over-expression, including increased tSC number, increased terminal boutons of the motor axon, and increased fragmentation of AChRs^25^. Furthermore, in electron micrographs (Fig. 4) we saw intercalation of tSC processes underneath the motor axon terminal and phagocytosis of the axon terminal by the tSCs, likewise seen in the NRG1-III over-expressers. Neuregulins are a family of growth factors that affect Schwann cell growth, proliferation, and myelination, among other functions, and NRG1-III is a membrane bound isoform that provides juxtacrine signals from axon to SC^38^. The NRG1 signaling pathway is essential in development, and although a full knockout of NRG1 is embryonic lethal, mice that are hemizygous for the null allele display numerous phenotypic abnormalities consistent with decreased axo-glial NRG1 signaling^25,32,39^. To determine whether the NRG1 signaling pathway is involved in the phenotypic changes seen at NMJs 3 weeks post-tSC ablation, we crossed an NRG1 null allele into the DTR expressing mouse line to generate “EXP, NRG1  +/−” mice. The “EXP, NRG1 +/−” mice were hemizygous for the NRG1 null allele, which affects all NRG1 subtypes in all tissues, but otherwise were genetically identical to the EXP mice. Three weeks after tSC ablation of EXP, NRG1 +/− mice, the number of tSCs (3.8 ± 0.19, Fig. 5A) was reduced to a level in between experimental and control animals and the number of terminal boutons (6.0 ± 0.19, Fig. 5B) was not significantly different from sham controls. The number of distinct AChR islands (6.9 ± 0.59, Fig. 5C) was not significantly different from either experimental animals or controls. Finally, the junctional area of the EXP, NRG1 +/− mice (875 ± 32 µm^2^, Fig. 5D) was unaffected by NRG1 signaling manipulation. These data suggest that the NRG1 signaling pathway mediates the increase in tSC and axon terminal bouton number seen 3 weeks after tSC ablation, but changes in the post-synaptic AChR aggregates likely involve other factors.

## Discussion

When terminal Schwann cells were ablated locally at the NMJ, tSC number and coverage of the NMJ decreased significantly within the first week. Though tSC number and coverage of the axon terminal remained significantly depleted by 2 weeks, major remodeling events within the NMJ were evident by 3 weeks post-ablation when tSCs returned to the NMJs. Notably, the average number of tSCs per NMJ increased to a significantly higher number than shams by the 3-week time point. At the same time, there was a significant increase in the number of axon terminal boutons and the number of AChR islands per NMJ, resulting in NMJs that resembled the “fragmented” phenotype.

Despite these dynamic changes, we did not observe wholesale denervation of NMJs. Taking into account previous research in tSC ablation of frogs, which resulted in axon terminals beginning to degenerate after 1 week^8^, and an adult mouse model of late-onset demyelination, which showed extensive morphological abnormalities of de-myelinated axons^40^, we hypothesized that axon terminals would degenerate a week after tSC injury when the axon terminals of these adult mice were made bare. From there, we hypothesized that surviving, more proximal SCs would halt the degeneration and lead the stump of the axon back to the NMJ to reinnervate it. In reality, however, the α-motor axon terminal as a whole survived long enough for SCs to replicate and restore their coverage of the terminal. The limit of an α-motor axon terminal’s ability to survive without tSC coverage in an adult mammal is an important avenue for future investigation.

Although SCs associated with other skeletal muscle nerves drove Cre, and therefore DTR, expression (Fig. 1 and Supplementary Figure S1), we did not analyze the potential effects of ablating SCs associated with other axonal populations besides α-motor axons, which may react differently to the ablation of their SCs. Furthermore, the SCs of these other peripheral nerves are a possible confounding variable in this system, as injury to an axon has been shown to cause a response in neighboring Remak SCs whose axon was not injured^41^ and damage to sympathetic neurons has been reported to affect the NMJ^42^.

The present study analyzed the morphology of adult NMJs after tSC ablation, but the physiological effects of tSC ablation on neuromuscular transmission in an adult mammal has yet to be thoroughly explored. Experiments conducted in frogs showed that neuromuscular transmission is unaffected immediately after tSC ablation, but within a week, deficits in numerous parameters appeared, including reduced miniature end plate potential (mEPP) frequency, although there was no change in mEPP amplitude 1 week after tSC ablation. Two studies have analyzed neuromuscular physiology after tSC ablation in mice older than 4 weeks of age. The first ablated tSCs via complement-mediated lysis and showed no change in neuromuscular transmission immediately after ablation, in agreement with experiments in frogs^10^. The second utilized tamoxifen inducible DTX subunit A to ablate SCs in 30 day old mice. The investigators found defects in compound muscle action potentials, mEPP frequency, and mEPP amplitude within 6 days of SC ablation^12^. Furthermore, tSCs respond to and modulate synaptic transmission, and it is possible that the absence of physiological defects immediately after tSC ablation in frogs and mice is the result of tSCs having the ability to both positively and negatively affect synaptic efficiency, thereby creating a “null” effect^4^.

The fragmented phenotype seen 3 weeks after tSC ablation has been observed in other experimental paradigms, most notably after myofiber damage, either intentional^16–19^, myopathic^15,43^, or through natural aging^20–23,44^. The fragmentation seen in this study, however, is not the result of myofiber damage, as evident in the fact that tSC ablation does not produce a significantly higher number of centrally nucleated myofibers, a common marker of recent myofiber regeneration (Supplementary Figure S4). A possible explanation for AChR fragmentation without myofiber damage can be gleaned from past literature. A classic study showed that when neuromuscular transmission is blocked in part of the axon terminal, the portion of AChRs where transmission is blocked disappears^45^, and a recent study in which SCs were ablated in adolescent mice suggested that defects in neuromuscular transmission precede morphological changes to the NMJ^12^. Additionally, vital imaging experiments tracking axotomized NMJs during re-innervation have shown that those pieces of axon terminal that lack tSC coverage quickly disappear along with their associated AChR clusters, and in this way, changes in tSC branching patterns produce corresponding changes in the receptor distribution at the synapse^46^. Taking these data into account, it is possible that axon terminals without tSC coverage experience a loss of function or activity, and the receptors beneath those dysfunctional terminals are taken back into the myofiber, creating newly distinct receptor islands. Furthermore, a loss of synaptic function in portions of the axon terminal may explain the reduced junctional area after 2 weeks; if the defunct axon terminals occur in the outer boundaries of the junction, the disappearance of those receptors would result in a smaller junctional area. Finally, the mechanical intercalation of tSC processes between the axon terminal and the secondary folds of the post-synaptic apparatus (Fig. 4) may cause the transmission block that results in the loss of receptor patches.

The stark similarities of NMJs 3 weeks after tSC ablation and those of NRG1-III over-expresser mice prompted us to examine mice in which NRG1 signaling was diminished by breeding a null NRG1 allele into our line of experimental animals. This constitutive knock down of NRG1 signaling, across all neuregulin1 isoforms in all tissues, was sufficient to cause near sham-level numbers of tSCs and WT numbers of axon terminal boutons in animals 3 weeks after tSC ablation. These data suggest that these two phenotypic changes are mediated through NRG1 signaling, although it is impossible to say which NRG1 subtype and which cell type is involved. NRG1 signaling has previously been shown to govern aspects of tSC activity at the developing NMJ: the NRG1-III over-expresser mice underwent synapse elimination at a faster rate than wild-type mice and NRG1 +/− mice underwent synapse elimination at a slower rate^25^. Moreover, the process of synapse elimination involves the phagocytosis of axon terminals^24^. Taking into account past research revealing the role of NRG1 signaling during synapse elimination, our results suggest the possibility that the increased numbers of tSCs and the reduction of axon terminal contact into numerous boutons 3 weeks after selective ablation may be a recapitulation of some of the NRG1-mediated functions of tSCs during the maturation of the NMJ. Interestingly, the reduction in NRG1 signaling had no effect on average junctional area across NMJs and the average number of AChR islands was not significantly different from either SHAM or EXP mice. This suggests that the mechanisms behind the remodeling of the post-synaptic apparatus may involve signaling other than the NRG1 pathway. One possible candidate can be found in mice with skeletal muscle-specific extracellular signal-regulated kinase 2 (ERK2) knockout^47^, which display fragmented AChR clusters in the absence of myofiber damage. ERK2 is a component of a mitogen-activated protein kinase module in mammalian cells and is important for muscle development and maintenance^48^, and may contribute to the appearance of the fragmented phenotype 3 weeks after tSC ablation.

Considering decades of studies manipulating both axons and SCs in developing and adult mice, it is clear that the interaction between SCs and their axons is different in adult animals versus developing animals. In the developing vertebrate, SCs and axons are co-dependent and cannot survive without the other, but sometime during maturation, possibly sometime after the fourth post-natal week^49^, that co-dependency is lost. In tadpoles and developing mice, the selective ablation of SCs causes the degeneration of the axon and prevents the maturation of the NMJ^8,11–13^. A pair of recent studies have made progress in determining the mechanisms by which embryonic synapse formation relies on the presence of SCs. It was shown that motor axons could maintain contact with postsynaptic muscle fibers in the absence of SCs if synaptic activity was inhibited^13^. Then, it was determined that SCs express the thrombin-inhibiting serpins C1 and D1, which suggested that SCs facilitate stability of synaptic connections by inhibiting muscle-derived thrombin, whose release depends on synaptic activity, during development^11^. Similarly, when developing NMJs are denervated, SCs undergo apoptosis^49,50^. In adult animals, SCs do not undergo apoptosis after denervation, but rather undergo a litany of well documented phenotypic changes^1,36,51–53^, although adult SCs have been reported to eventually die when deprived of axonal contact for several months^54^. When adult motor axons are deprived of their tSC coverage, there is no immediate effect on neuromuscular function^8,10^, but in this study we have observed the long-term consequences of tSC ablation at young adult (2–6 month old) NMJs. We have shown that tSC ablation does not lead to the wholesale degeneration of α-motor axons, so it is possible that they are more resilient to the loss of SCs in the adult than they are during maturation. The ways in which axon-SC interactions differ between development and adulthood, and precise mechanism by which this switch from developmental co-dependency occurs, are compelling topics for further research.

Our model for the process by which the neuromuscular junction recovers from tSC death is as follows: after tSCs ablation at an NMJ, the α-motor axons initially are largely unaffected, either morphologically or functionally. After the first post-injury week, portions of the axon terminal either degenerate slightly away from the AChRs and/or there is a disruption in neuromuscular transmission along portions of the axon terminal. Either way, this causes receptors that no longer have functional neuronal input to be absorbed back into the myofiber and disappear from the NMJ along with those portions of the axon terminal. Between 2 and 3 weeks, operating through the neuregulin 1 signaling pathway, the new tSCs repeat aspects of their developmental role at the synapse. They proliferate, reclaim their role of capping the axon terminal, and intercalate processes between the axon terminals and the muscle fiber, thus causing the now denervated patches of receptor to be absorbed into the myofiber, creating new islands of receptor synapsing with axon terminal varicosities connected by thin axonal processes.

## Supplementary information

Supplementary file1 (PDF 440 kb)

## Data Availability

All data presented in this article are available from the corresponding author upon reasonable request.
