# Supplementary material for: Morphological remodeling during recovery of the neuromuscular junction from terminal Schwann cell ablation in adult mice
Source: Sci Rep. 2020 Jul 7;10:11132. doi: 10.1038/s41598-020-67630-1 (PMC7341867; doi:10.1038/s41598-020-67630-1)
Supplement: Supplementary file 1 — Supplementary file1 (PDF 440 kb) [file 41598_2020_67630_MOESM1_ESM.pdf]

Morphological remodeling during recovery of the neuromuscular junction from terminal Schwann cell ablation in adult mice

Robert Louis Hastings[1,2,†], Michelle Mikesh[3], Young il Lee[2], Wesley J. Thompson[1,2,*]

[1]Texas A&M Institute for Neuroscience, Texas A&M University, College Station, TX, USA
[2]Department of Biology, Texas A&M University, College Station, TX, USA
[3]Center for Biomedical Research Support (CBRS), University of Texas at Austin, Austin, TX, USA

[†]Correspondence: robertlouishastings@gmail.com
[*]Deceased March 26, 2019

Supplementary Figures

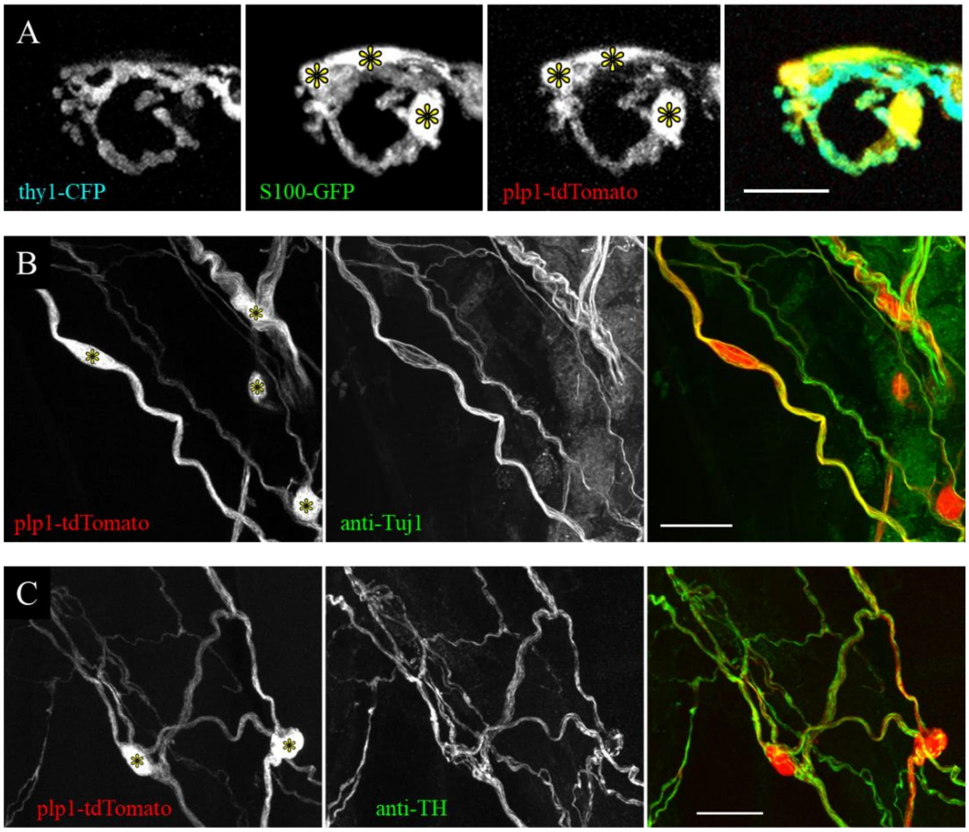

Supplementary Figure S1. The plp1-Cre^{ERT} transgene drives Cre activity in Schwann cells throughout skeletal muscle tissue. (**A**) Young adult STM NMJ expressing thy1-CFP, S100-GFP ("Kosmos"), plp1-Cre^{ERT}, and Ai14 transgenes. Expression of plp1-Cre^{ERT} transgene (plp1-tdTomato) at the NMJ faithfully replicates the labeling pattern of the "Kosmos" S100-GFP transgene. (**B**) plp1-Cre^{ERT} transgene labels Schwann cells outside of the endplate band in a soleus muscle. Anti - β-III Tubulin (Tuj1) staining indicates that the Cre-expressing extra-junctional cells are associated with axons. (**C**) SCs in soleus muscle tissue outside of the endplate band. The plp1-Cre^{ERT} transgene labels Schwann cells that are associated with sympathetic axons, stained with an anti-tyrosine hydroxylase antibody. Fiji and Microsoft Powerpoint were used to generate this figure. All scale bars 20 μm.

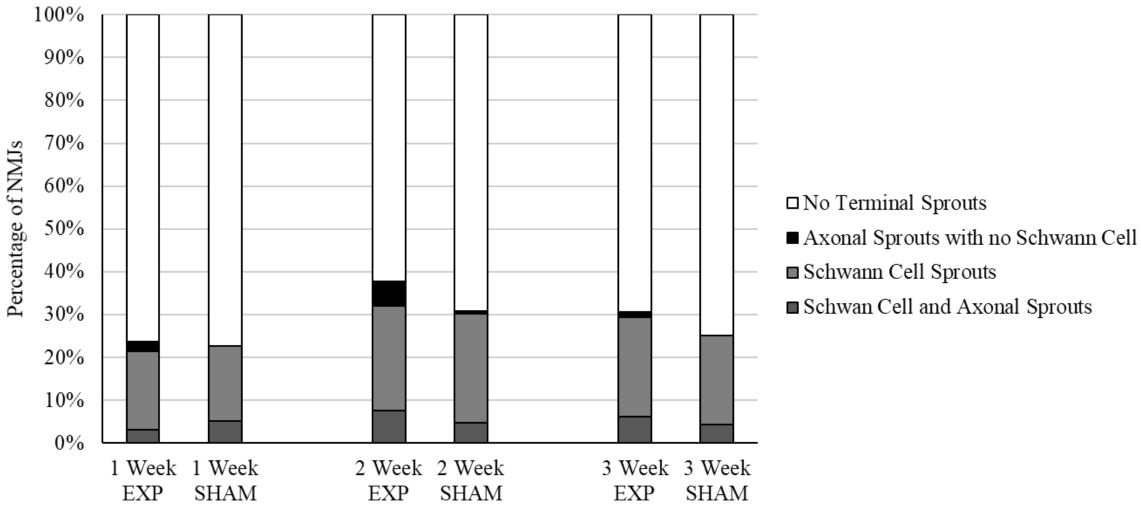

Supplementary Figure S2. Terminal sprouting is not a significant feature of NMJ recovery after tSC ablation. We analyzed terminal sprouting from NMJs, which is known to occur during damage to the motor axon and with NRG1-III overexpression [25, 46, 50, 55, 56]. At no time point post-tSC ablation was there a significantly different distribution of terminal sprouts compared to sham animals, with roughly 70% of NMJs across each time point showing no terminal sprouts. An interesting phenomenon seen in the tSC ablated mice was axon terminal sprouts with no tSC process covering it that could be resolved or viewed with epifluorescence, which peaked in prevalence two weeks after tSC removal. It is extremely rare, though not unheard of [46], for there to be axonal processes without tSC coverage, but those processes likely degenerate quickly without tSC coverage. In our results, only 1 in 300+ NMJs from our sham animals displayed such a process. There were several bare axonal processes that extended beyond the BTX stain in the tSC ablated animals, which suggests the possibility that they may be the remains of degenerating axonal processes after their associated tSC died from DTX exposure.

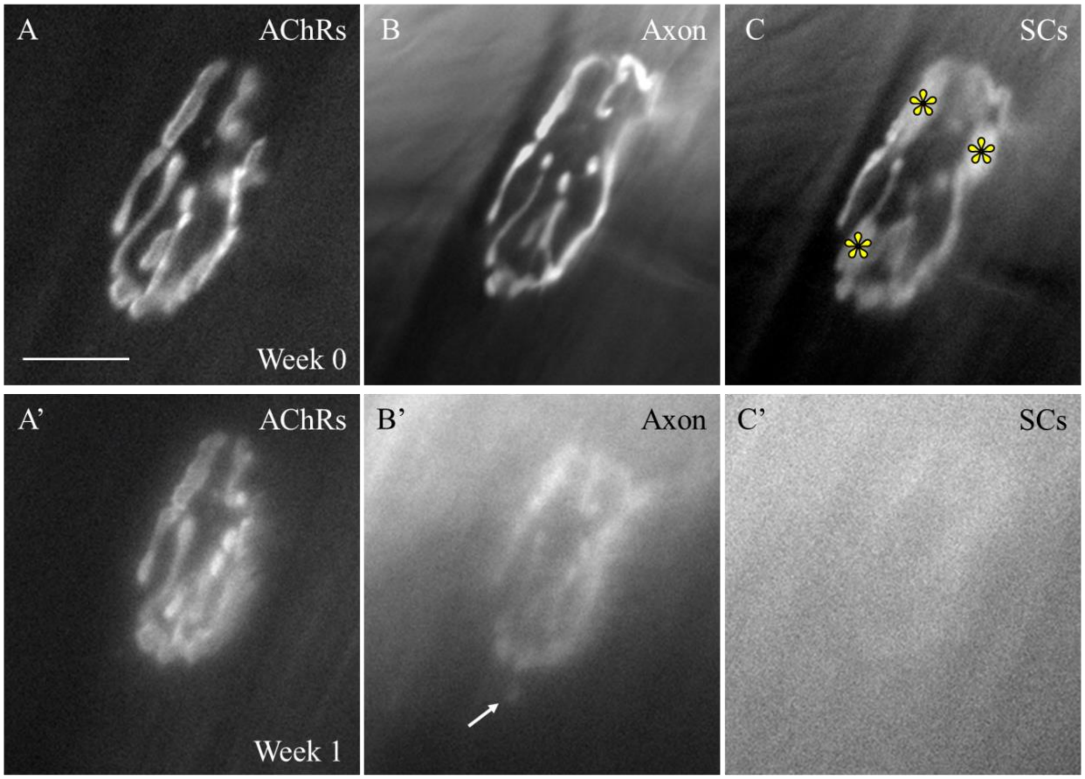

Supplementary Figure S3. Repeated vital imaging confirms that axons remain after tSC ablation. NMJs were imaged in live mice before DTX application (Week 0) and weekly after DTX application. Shown here is the same NMJ before and one week after tSC ablation. Note that the AChR stain (**A** and **A'**) and the transgenic axonal label (**B** and **B'**) do not undergo major morphological changes, except for what appears to be a small axonal sprout (arrow) which developed after tSC ablation. The transgenic SC label, however disappears entirely. Initially, there are three tSCs (asterisks) that completely cover the axon terminal (**C**). One week after SC ablation, however, there is no detectable transgenic tSC label (**C'**). At two and three weeks post-tSC ablation, we were unable to re-locate NMJs that had lost tSC coverage at one week, presumably because those NMJs, and their neighbors typically used for the relocation of NMJs, had been remodeled to the point of being unrecognizable. Fiji and Microsoft Powerpoint were used to generate this figure. Scale bar 20 µm, applies for all panels.

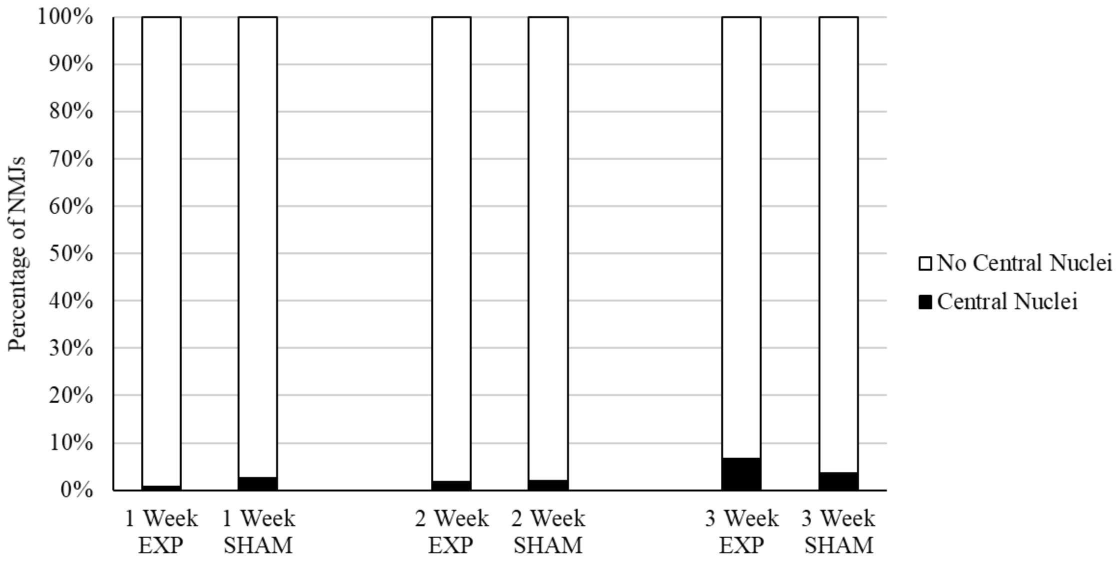

Supplementary Figure S4. NMJ remodeling is not caused by myofiber damage. To determine whether the observed fragmented phenotype was the result of myofiber damage, we recorded whether the NMJ being measured was associated with a myofiber containing a string of central myonuclei near the junctional region, which are well understood to appear after damage to a myofiber. We determined that there was no significant difference between the distribution of NMJs that were associated with central nuclei chains between shams and controls at any time point, therefore suggesting that the changes observed at the NMJ were not the result of incidental myofiber damage from the surgery.

Supplementary References

55. Kang, H., L. Tian, and W. Thompson, *Terminal Schwann cells guide the reinnervation of muscle after nerve injury.* Journal of Neurocytology, 2003. **32**(5-8): p. 975-985.

56. Trachtenberg, J.T. and W.J. Thompson, *Nerve terminal withdrawal from rat neuromuscular junctions induced by neuregulin and Schwann cells.* Journal of Neuroscience, 1997. **17**(16): p. 6243-6255.
